# Vaginal Discharges in Young Females

**Published:** 1851-09

**Authors:** 


*Vaginal Discharges in Young Females.*— There are, we presume, but few practitioners who have not on some occasion or other, been consulted by an anxious and distressed mother, in relation to the existence of a vaginal discharge in her daughter; and all must confess to the very delicate and responsible situation in which one finds himself placed, when he is expected to give an honest and satisfactory answer — honest, one based upon true science and careful investigation; satisfactory, one that will dispel the cruel and dark cloud of mystery and suspicion that lowers over the fair fame of one, who is as yet but an infant. What anguish does not one unguarded word of the physician plant in that mother's heart? It casts a veil of sorrow over the cherished flower, that years, aye, even, deep solicitude and maternal affection cannot rend.

Of the several cases which have fallen to us in the course of practice, we will relate but one, which we trust will prove a lesson for those who are too *old* to learn, but not too *wise* to give bad advice.

Sometime during the past year, we were waited upon by a highly respectable and intelligent lady from W——, a distance of 120 miles from this place, and our advice requested in relation to some peculiar ailment, under which her daughter, aged eight years, had been laboring for several months. With much reluctance, she had consulted one physician, and then another in W——, and instead of encouragement and hope, they had crushed her by aspersing the reputation of the daughter. And to convince the mother of the correctness of their position, they urged upon her to use mild and persuasive measures, and if these should prove of no avail, to resort to threats and punishment, in order to extort from the child the avowal of guilt. In spite of repeated harsh measures, the daughter remained firm in the assertion of her innocence; and obtaining neither satisfaction to her mind, nor relief to the child's complaint, she was induced to visit Plattsburgh.

We will not attempt to portray the feelings of the mother when she unfolded her tale of affliction; but we were happy in promising the fulfillment of her most sanguine wishes,— the return of her daughter to her affections, pure and virtuous. After a close examination, we learned from the lady, that at different times she had noticed something like "pieces of white thread" in the evacuations of her daughter. The mystery was at once solved, and the ignorance of the two physicians made clear and manifest. We prescribed a mixture of castor oil and spirits of turpentine, and also ordered the application of the turpentine, so as to produce a slight degree of irritation about the verge of the anus. On the second day, the discharge had sensibly diminished; *ascarides* in large quantities were discovered in the fæces, and on the sixth day, a disease of seven months' duration, and which was rapidly undermining the child's frail constitution, was promptly and successfully eradicated by this simple treatment.

Comment upon the wisdom and *certainty* of the two medical advisers is unnecessary; their acquirements were appreciated at their full value by that mother, her friends and the public, for be it known, that base to their professional honor, they had talked of the case in their respective circles of acquaintance.

It should also be borne in mind, that a high degree of importance is attached to the true diagnosis of vaginal discharges, in a medico-legal point of view. We might report many instances of the disastrous and heart-rending consequences resulting from ignorance in these cases; but as this subject will be fully considered in the department of Medical Jurisprudence of our ensuing number, we will only add, that the physician cannot be too minute in the examination of the causes, and sacredly guarded in the diagnosis and prognosis of vaginal discharges. — *Northern Lancet, (Editorial.)*

